# Gaining Empathy for the Learner: A Way to Identify Unique Themes and Patterns in Medical Student Experiences

**DOI:** 10.1007/s40670-025-02547-w

**Published:** 2025-10-28

**Authors:** Kristin Machac, Andrew Binks, Patrick Bonson, Renée LeClair

**Affiliations:** 1 MKM Global Consulting Inc., Roanoke, VA USA; 2 Present Address: Department of Basic Science Education, Virginia Tech Carilion School of Medicine, Roanoke, VA USA; 3 Virginia Tech Carilion School of Medicine, Roanoke, VA USA; 4 Department of Physical Therapy, Radford University, Roanoke, VA, USA

**Keywords:** Experience diagrams, Design thinking, Program evaluation, Ethnographic interviews

## Abstract

A variety of program evaluation tools are used in medical education, including grade-based, narrative, and quantitative methods. While providing valuable feedback, they may not fully capture the lived experiences of the learner. To enhance understanding and empathy for the medical school experience, we conducted ethnographic interviews of learners across all years of training. Emergent themes were compared with feedback from traditional instruments. Our empathetic methods not only confirmed themes identified through conventional evaluations but also revealed additional factors that heavily impacted learners. This highlights the need for empathic, human-centered approaches to generate a holistic picture that better informs programmatic change.

**Supplementary Information:**

The online version contains supplementary material available at 10.1007/s40670-025-02547-w.

## Background

Medical education institutions routinely gather data about learners’ perceptions and experiences of learning [1] through end of course evaluations [2, 3] to guide institutional decisions regarding curricular changes and budget allocations [4]. These traditional evaluation methods, however, may fail to provide insights into how learners feel about their experiences and communities of learning [5]. Design thinking (DT) research [6] may have an important role in the evaluation process to bridge the gaps left by traditional methods.

DT is a human-centered, problem-solving approach with an emphasis on understanding user needs and developing innovative solutions. It includes five phases, the first of which is beginning to empathize. Empathic evaluation methods aim to understand people’s experiences, emotions, motivations, and needs by seeing the world from their perspective [7]. In medical education, there are examples of using ethnographic interviews [8–10] to understand the experiences of individuals in the clinical setting [11] or in response to peer education [12]. Integrating such a holistic approach into program evaluation may provide a more comprehensive understanding of the learner experience not provided by traditional methods and therefore better inform programmatic change. For example, this holistic and empathic approach to identify factors that influence learner experience may capture influences that originate from outside the classroom and are undetected by traditional methods. A more comprehensive insight reduces the risk of episodic or reactionary curricular changes that can occur without a complete understanding of learner concerns and their evolution across their 4-year journey. We therefore wished to compare learner perspectives from empathic methods with traditional course evaluations as a process for informing change.

Our aim was to collect learner-driven, empathetic insights to synthesize a collective, longitudinal learner experience to identify opportunities for future curricular transformations. Unlike previous studies that employed empathetic approaches in the context of medical curricula and professional climate [13, 14], we compared these methodologies with traditional qualitative evaluation methods to evaluate the similarities and differences in the experiences that the two approaches captured.

## Activity

**Fig. 1:**
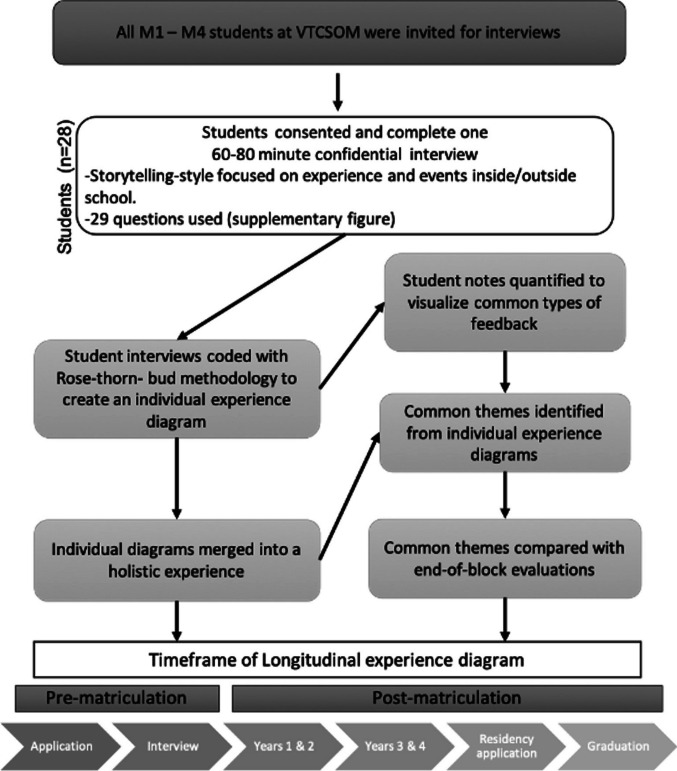
Summary of methods used and the flow of data. First (M1) thorough fourth (M4) medical students were invited to participate in a confidential interview that focused on 29 questions to allow for a story-telling process. During the interview, students’ responses were coded using a rose-thorn-bud method and used to create an individual experience diagram. Individual notes were used to inform common themes and merged to create a longitudinal experience diagram that included responses from interviews across all years. Common interview themes were compared to themes reported from VTCSOM end of block (EOB) evaluations

First through fourth year students (*n*=28) at Virginia Tech Carilion School of Medicine (VTCSOM) were interviewed by a certified facilitator and instructor at the LUMA Institute using a set of 29 questions modified from standard questionnaires [15]. The questions were used to prompt semi-structured storytelling and engender elaboration of specific stories (Supplementary Figure 1). Participation in the interview was voluntary (VT IRB protocol #19–400). Participants were asked to use the rose-thorn-bud method to codify whether reported experiences were positive, negative, or an area for improvement, respectively. Opportunity to confirm or revise the codification was offered at the end of the interview. The responses were summarized in an experience diagram consisting of brief researcher annotations codified with the rose-thorn-bud [16] approach to denote the type of experience the learner had. Affinity clustering of the rose, thorn, bud findings identified common categories and overarching themes of experiences across the curricular timeline [17]. Interview comments from M1–M4 learners were distributed along a four-year time frame to generate a longitudinal experience diagram aligned with the VTCSOM curriculum (Fig. 1).

Themes identified in VTCSOM EOB evaluations were collated from the same academic years that interviews were conducted (2018–2019 and 2019–2020) for comparison with themes from the empathic interviews. Thematic coding of the course evaluations was performed by the VTCSOM Office of Evaluation and Assessment, who define a theme as four or more open comments about the same topic. Themes from interview data were identified using the same method by two independent individuals.

## Results and Discussion

Comparison of the longitudinal experience diagram and rose-bud-thorn themes, with themes identified in course level evaluations illustrated both similarities and differences in reported experiences, highlighting the need for, and utility of, these complementary tools. While the end of course evaluations captured narrow themes that were largely specific to either a course or an academic year, the empathic interview approach also provided learner-centered comments reflecting externally related themes that were not detected by the traditional evaluation (see Table 1). Of the ten themes identified by learner interviews, seven pertained to the learning environment (i.e., not course specific) such as student support resources, study styles, and class cohesion.

**Table 1:** Comparison of themes represented in interviews when compared to end of block evaluations. Astrix indicates themes common to both methodologies

Themes from student interviews	# of individual comments per theme	% of total comments	Themes from VTCSOM End of Block Evaluations	# of individual comments per theme	% of total comments
Basic science domain*	156	13	Clinical science domain*	308	12.9
Problem-based learning (PBL)*	117	9.8	Basic science domain*	299	12.5
Orientation week	68	5.7	Anatomy	194	8.1
M3 fall	54	4.5	Interprofessionalism	171	7.1
Student support	52	4.3	Problem-based learning (PBL)*	156	6.5
Personal study style	51	4.2	Research domain	140	5.8
Clinical science domain*	49	4.1	Standardized patients	107	4.5
Cohort cohesiveness	49	4.1	Ultrasound	54	2.2
Personal backgrounds	46	3.8			
Diversity	39	3.3			
Total number of comments collected		1198	Total number of comments collected		2394

The overlap in themes generated by the two evaluation methods pertained to the three VTCSOM curricular domains (problem-based learning (PBL), basic science domain, and clinical science domain). These themes likely arose in both data sets because learners spend the most time in these activities [18] and suggest that despite the differences in these tools and sampling methods, there is some consistency in the learner perspectives gathered. Comparing the percentage of rose, bud, thorn, and “other” comments gathered by both methods, there was no significant difference in buds (*p*=0.115, *T*-test) or roses (*p*=0.146) between EOB and DT. Thorns, however, were significantly more prevalent in EOB vs. DT (31.9% ± 7.6 vs. 23.5% ± 2.5 respectively, *p*=0.011, T-test), while “other” comments were less prevalent in EOB vs. DT (7.9% ± 4.9 vs. 2.1% ± 5.3 respectively, *p*=0.026, *T*-test). Learners were less likely to be negative (thorns) in the empathic approaches and more likely to provide opportunities (buds) for improvement during their interview. This may be due to the face-to-face nature of the interview process generating a sense of accountability that did not arise to the same level in the anonymously completed EOB process.

The differences in learner responses demonstrated some advantages of the student-centered interview approach for operationalization. For example, themes surrounding curricular organization in the basic science domain arose as an opportunity (bud) in both interview and EOB evaluation data; however, learners’ comments from the EOB evaluations were generally static (e.g., “needs improvement” or “could be better organized”) and did not include rationalization or suggestions for improvement. Although this static information provides a starting point for change, its shallower nature can lead to modifications that fail to address the learners’ fundamental concerns. In contrast, the more comprehensive empathic evaluation provided descriptive ideas of how curricular organization could be improved. Participants’ comments were more specific, “[they] don’t finish their slides, so I don’t know what is important’ or ‘more case-based teaching is helpful.” These are more targetable opportunities that could produce responses aligned with end-user needs that could be used to inform a more focused solution.

**Fig. 2:**
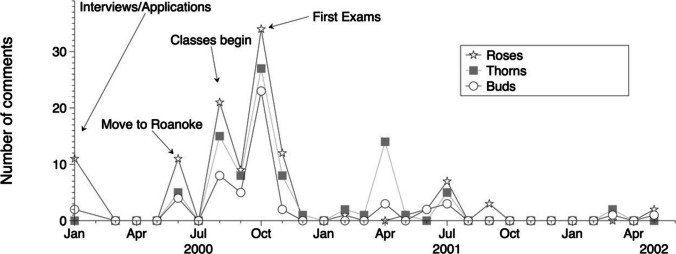
Frequency of rose-thorn-buds across a time continuum. Number of comments increased around times of change

Frequency of rose-thorn-buds tended to cluster around times when learners transitioned curricular formats or elements that they had not previously experienced (i.e., periods of change); this may partially explain the concentration of rose-thorn-buds relating to the first year of medical school (Fig. 2). For example, most comments clustered around the medical school application process, moving to campus, the start of classes, and the first round of medical school exams. Although it is important to reflect on experiences throughout a medical student’s career, we postulate that experiences prior to matriculation and within the first months of interaction with a program may disproportionately shape a learner’s perspective of the four-year experience.

Previous studies have suggested that medical students perceptions of the learning environment shift over time [19] and that a positive learning environment contributes to better performance [20]. While course level evaluation is essential, it may be more impactful if coupled with a complementary tool such as this presented here to address climate vs. course level changes [21, 22]. By interviewing learners across M1–M4, we have gathered a more complete perspective of their experience which may inform more longstanding change when considered alongside course level modifications [23].

Although the results of this study are unique to our program, our process highlights the advantages of empathic evaluation and how it can serve in a complementary role in qualitative program evaluation. We have identified key areas to gather information that could easily be adopted by other institutions, and the visual illustrations of the learners’ lived experiences afford an enhanced opportunity for stakeholders to align and collaborate on future opportunities for improvements. In comparison with traditional program evaluations, we have illustrated that these interviews can capture similar themes, include more impactful external influences, and provide a mechanism for more constructive and less negative feedback.

Although we observed significant advantages of using an empathetic process to complement traditional program assessments, it is not without its challenges. The first being its time and resource-intensive nature, but we saw opportunities to streamline the process in the future. Future students could be prompted to codify their own responses based on established experience diagrams or themes utilizing online whiteboards as a form of continual “member-checking” that would drastically reduce time to collect the data while keeping the themes current. This would also be an opportunity to address the potential selection bias that may be due to the limited number of initial interviewees. The addition of a member-checking process by a larger group of learners assessing initial interview summaries would provide additional confidence that they were representative of the whole learner population.

Despite these challenges, this work represents a potential new application of empathic research to gain a better understanding of the medical student perspective, allowing us to broaden our understanding of the currently collected metrics and formulate a more pertinent and focused response.

## Supplementary Information

Below is the link to the electronic supplementary material.

Supplementary figure 1: Questions utilized for student interviews. These are open ended to encourage a storytelling format (PNG 1.21 MB)
