# Supplementary figures and images for: Gaining Empathy for the Learner: A Way to Identify Unique Themes and Patterns in Medical Student Experiences
Source: Med Sci Educ. 2025 Oct 28;36(1):117–21. doi: 10.1007/s40670-025-02547-w (PMC13043984; doi:10.1007/s40670-025-02547-w)

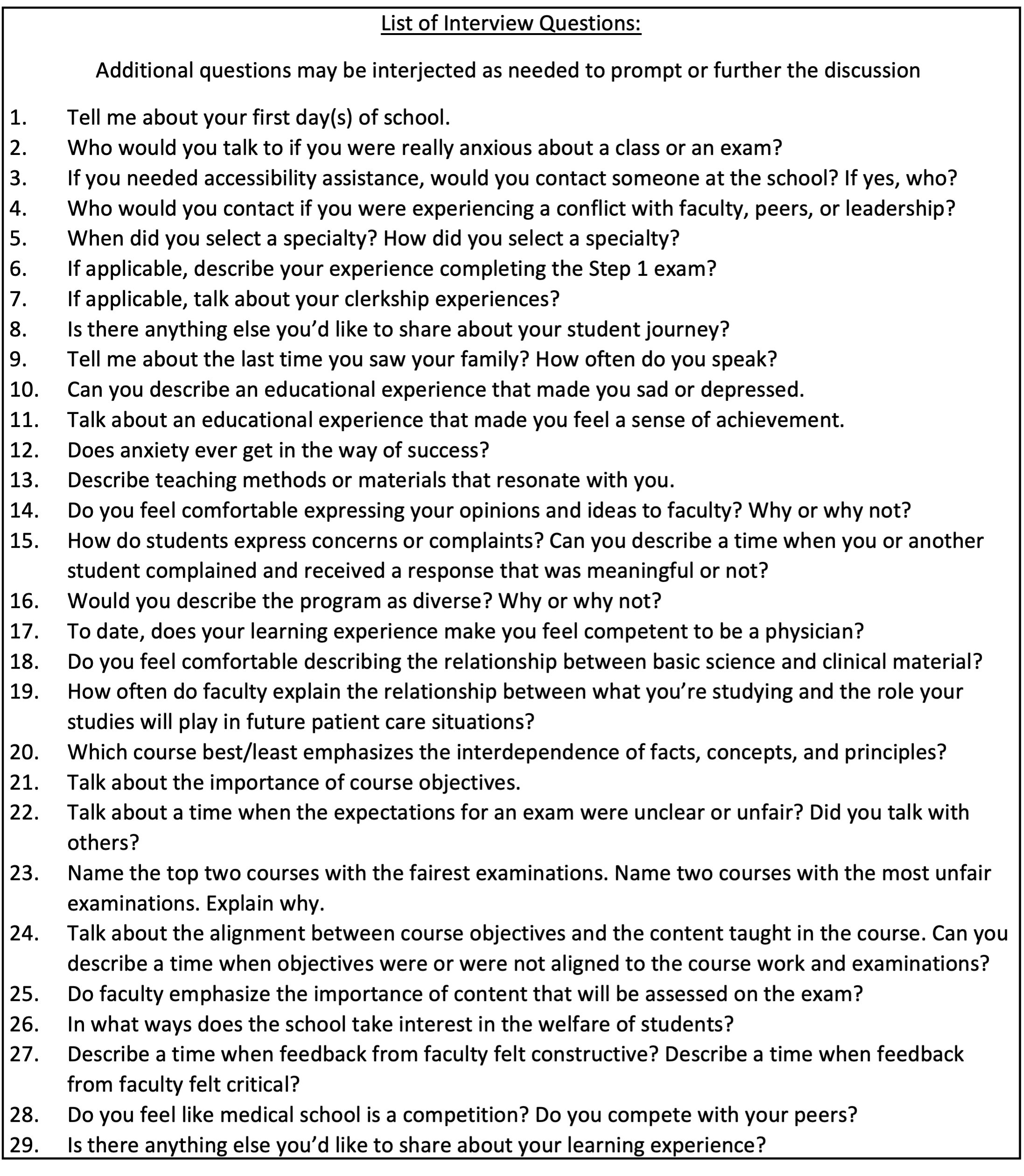

Supplement: Supplementary file 1 — Supplementary figure 1: Questions utilized for student interviews. These are open ended to encourage a storytelling format (PNG 1.21 MB) [file 40670_2025_2547_MOESM1_ESM.png]
